# A formalism and methodology for measurement and control of LINAC isocenter

**DOI:** 10.1002/acm2.13981

**Published:** 2023-04-03

**Authors:** Nicholas G. Zacharopoulos, David A. Fenyes

**Affiliations:** ^1^ Aktina Medical Corp. Congers New York USA

**Keywords:** isocenter, radiotherapy, Winston–Lutz

## Abstract

**Background and Purpose:**

Despite the acknowledged need for a stable reference point for LINAC isocenter quality assurance (QA), no standard for such a reference point has been established. This paper introduces a practical and robust technique for measuring and tuning LINAC isocenter within a stable reference frame based on the collimator axes of rotation.

**Methods:**

We develop a framework based on *physical isocenter*, a refinement of the approach by Skworcow et al. The physical isocenter provides a relatively stable, first principles spatial point from which other LINAC parameters can be referenced. An optical tracking system was used to measure the collimator axes with high precision and an isocenter cost function was implemented to ensure a unique isocenter location. The same optical tracking system was used to (a) align the couch axis to the physical isocenter, (b) align the radiation beam to the collimator axes, and (c) position a marker precisely at the physical isocenter to demonstrate the effectiveness of the approach.

**Results:**

The framework was successfully demonstrated on an Elekta LINAC. The physical isocenter was shown to be repeatable with a standard deviation of 0.03 mm for the position and 0.03 mm for the radius. The couch axis was aligned to physical isocenter within 0.07 mm. The average collimator to beam axis distance before beam alignment was 0.19 and 0.10 mm after. All these steps were performed within 3 h, showing that the method is efficient when applied to isocenter optimization. The time required to measure physical isocenter and guide a marker to it for day‐to‐day isocenter QA was under 10 min.

**Conclusions:**

We have presented a modular, practical framework for isocenter characterization and optimization based on *physical isocenter*, which is a stable and fixed reference point.

## 1 INTRODUCTION

The American Association of Physicists in Medicine (AAPM) Practice Guideline for LINAC quality assurance (QA)^1^ recommends establishing a reference frame for finding isocenter for routine linear accelerator (LINAC) QA. An isocenter reference frame that allows for reproducible Winston–Lutz (WL) marker positioning would enable more precise root cause analysis when LINAC isocenter geometric inaccuracies arise.

The collimator axis of rotation is an excellent candidate for establishing such a frame of reference because it is mechanically stable, clinically relevant, and amenable to measurement using simple methods. Skworcow et al.^2^ proposed a version of mechanical isocenter based on the collimator axes. They used an optical tracking system to estimate three‐dimensional representations of the collimator axes at multiple gantry positions and derived an isocenter that minimized the maximum isocenter‐to‐collimator axis distance. This isocenter based on collimator axes was compared to a radiation isocenter derived using an analogous 3D technique based on WL images at different distances from the target. The use of a single fixture for optical and WL imaging unified the optical and WL coordinate systems.

Chojnowski et al.^3^ developed a radiation‐based construction of mechanical isocenter based on the collimator central axis, and measured radiation isocenter in reference to this constructed mechanical isocenter, following the approach or Skworcow et al.^2^ Again, a specialized fixture was used to ensure collimator axes and WL data shared the same coordinate system.

By constructing a version of mechanical isocenter based purely on the 3D collimator axes, and a corresponding version of radiation isocenter based on the 3D beam axes, Skworcow et al.^2^ and Chojnowski et al.^3^ created a reproducible reference frame based on LINAC mechanical properties, to which the radiation isocenter can be tuned by reducing beam alignment errors due to focal spot offset. Their techniques, however, relied on the construction of measurement fixtures that required knowledge of the location of the collimator axis of rotation. Skworcow et al.^2^ created a fixture with two optical tracking markers visually aligned with the collimator's axis using the collimator crosswires. The fixture used by Chojnowski et al.^3^ contained multiple levels of markers that required symmetric placement about the collimator's axis. Although corrections factors were implemented to compensate for the unavoidable assembly inaccuracies, a more robust method would not require any pre‐existing knowledge of the collimator axis that the method is purporting to measure.

We present here a practical method for establishing a reference frame for LINAC isocenter tuning and verification, based on a more robust determination of the three‐dimensional collimator axes of rotation, building on the work of Skworcow et al.^2^ and Chojnowski et al.^3^ Our approach defines a fixed reference frame without the use of radiation and allows for the precise placement of a WL marker within that reference frame. The framework is robust in that it allows for both the couch axis of rotation and for beam alignment to be independently optimized.

## 2 METHODS

### 2.1 Physical isocenter

A reference frame for isocenter based purely on the collimator axes of rotation has the advantage of being largely invariant to the various LINAC subsystems such as couch, beam alignment, and field shaping that are likely to drift. This approach, suggested by Skworcow et al.,^2^ uses a definition of mechanical isocenter that minimizes the maximum distance between mechanical isocenter and all collimator axes.

**FIGURE 1:**
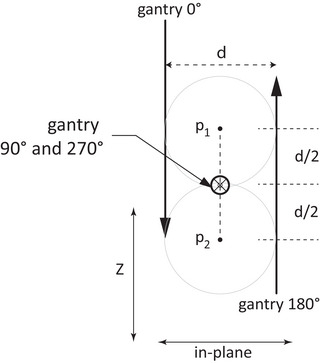
An example of an underdetermined minimax system of collimator axes of rotation at the four cardinal gantry angles (the 270° and 90° axes have directions in and out of the page, respectively). As is often the case, the 0° and 180° axes are spread in the in‐plane direction due to gantry sag, while the 90° and 270° axes are coincident and well centered. Any point along the segment between *p*_1_ and *p*_2_ will satisfy the minimax condition, with a maximum error equal to half the gantry spread distance *d*.

The mechanical movement of the gantry, however, may create a set of collimator axes for which a minimax definition fails to yield a unique solution. This is demonstrated in Figure 1, which shows a typical configuration of LINAC collimator axes for the cardinal gantry angles. The in‐plane gantry shift spreads the 0° and 180° collimator axes, producing a lower bound on the minimax error. As a result, the minimax condition proposed by Skworcow et al.^2^ is satisfied by a set of points along the line from *p*_1_ to *p*_2_. All solutions between these two points will yield the same minimum isocenter radius of *d*/2.

We, therefore, must include additional constraints to ensure a unique solution. For this work, we want the collimator‐based isocenter to minimize not only the maximum axis error, but also the overall errors across all clinically relevant gantry angles. To this end, we define it as the point that minimizes a cost function with a term for the maximum axis error, and a term for the root‐mean‐square axis error. Both terms are weighted equally, according to the cost function:

(1) $$\begin{array}{c} C\left(p\right)=\max_{\theta \in \{\theta_{1},\text{…},\theta_{n}\}}∥\delta \left(p,A_{\theta }\right)∥+\underset{\theta \in \{\theta_{1},\text{…},\theta_{n}\}}{\mathrm{rmse}}∥\delta \left(p,A_{\theta }\right)∥ \end{array}$$

where $\delta (p,A_{\theta })$ is the error vector for a collimator axis $A_{\theta }$ (at gantry angle θ) and isocenter position *p*. The cost function is evaluated over the set of *n* axes.

The error vector $\delta (p,A_{\theta })$ in Equation (1) represents the shortest distance from the isocenter point *p* to a collimator axis $A_{\theta }$:

(2) $$\delta \left(p,A_{\theta }\right)=\vec{p-p_{c}}$$

where $p_{c}$ is the point on $A_{\theta }$ closest to *p*.

To avoid confusion, we refer to the collimator‐based isocenter described above as the “physical isocenter.” This will differentiate it from the “mechanical isocenter,” which is typically defined as the intersection of some combination of the gantry, collimator, and couch axes of rotation.

### 2.2 Solving for physical isocenter

Because there is no closed‐form solution to the physical isocenter definition presented above, a solution must be determined using optimization search techniques. We used the Nelder–Mead downhill simplex method,^4^ which is well suited to optimization problems that may not be differentiable everywhere (for example, at isocenter). The starting point was set to the centroid of the set of points of closest approach between all axes pairs that met the criteria of having at least a 45° separation between them. The chosen termination criteria were a minimum simplex size of $1\times 10^{-3}$ mm.

### 2.3 Collimator axis determination

The basic component of the physical isocenter determination, as described above, is an accurate measurement of the collimator axis of rotation. We used a prototype version of the isoPoint optical tracking system (Aktina Medical Corp., Congers, NY, USA) to measure the collimator axes. At the time of data collection, this system was not yet commercially available.

**FIGURE 2:**
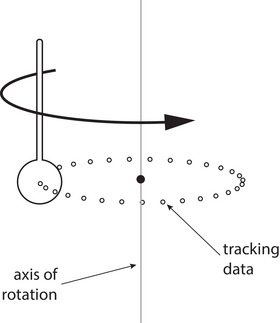
Schematic of the method used to determination the collimator axis of rotation. An optical tracking marker is rigidly connected to the LINAC collimator. The marker path through space as the collimator rotates is determined via optical tracking and used to compute the axis of rotation of the marker (and the collimator).

A stereo‐vision camera pod is positioned on the couch in front of isocenter and a tracking tool is attached to the collimator. The schematic in Figure 2 demonstrates this process. The tracking tool consists of an optical marker rigidly connected to the LINAC collimator so that it rotates with the collimator through a circular path.

As the collimator and marker rotate together, the isoPoint software, which is connected to the camera pod, uses optical tracking to determine the three‐dimensional location of the marker at various points along its path. From these locations, the software then computes the axis of rotation of the marker (and therefore the collimator).

To determine the collimator axis of rotation from the set of three‐dimensional tracking tool positions, the points are first projected onto a best‐fit plane determined by singular value decomposition. From the points projected in the best‐fit plane, a hyper least‐square circle fit^5^ is then used to determine the axis location within the best‐fit plane. The 3D axis of rotation is computed as the line passing through the center of the circular fit perpendicular to the best‐fit plane in the original coordinate system.

### 2.4 iMarc dataset acquisition

**FIGURE 3:**
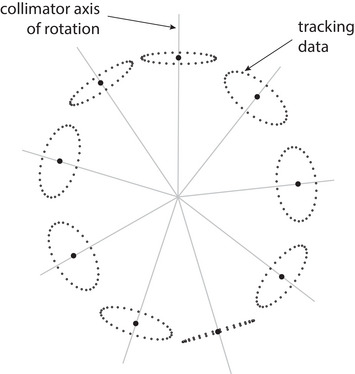
The iMarc (isoPoint multi‐axis rotation center) dataset includes several collimator axes of rotation at different gantry angles.

The physical isocenter is computed from multiple collimator axes of rotation acquired at different angles through the gantry's full range of motion. We refer to this dataset as the iMarc (isoPoint multi‐axis rotation center) dataset, shown in Figure 3. At a minimum, the cardinal gantry angles should be included. Each axis must be acquired without disturbing the stereo‐vision camera pod location so that all axes share the same coordinate system. The iMarc dataset is used as the input to the cost function in Equation (1), which is minimized via optimization search to determine the physical isocenter location.

### 2.5 Beam alignment

The physical isocenter defined in Section 2.1 is based purely on the collimator axis of rotation, which is an idealized surrogate for the radiation beam. This allows the physical isocenter to be independent of beam misalignment due to focal spot offset. This approach can only be useful, however, if the radiation beam can be aligned with the collimator axis of rotation. Once the beams have been aligned across all gantry angles, the radiation isocenter will converge to the physical isocenter.

Practical techniques for measuring and reducing the focal spot offset to align the radiation beam with the collimator axis include the corotational penumbra modulation^6^ as well as techniques that contain radiopaque markers arranged at different distances from the target to deduce the beam angle relative to the collimator axis.^7^, ^8^ Phantomless techniques based only on beam symmetry have been proposed but can hide the relative contribution of focal spot position and beam angle.^9^, ^10^

By contrast, we present a technique that computes the offset based on the difference between the expected and found marker position within a transmission image, for a single marker placed along the collimator axis of rotation. This technique has the advantage of harmonizing with the currently presented workflow without requiring an additional phantom as the method of Slama et al.,^7^ and is independent of the distance between the MLC and jaws, unlike the method of Chojnowski et al.^10^

**FIGURE 4:**
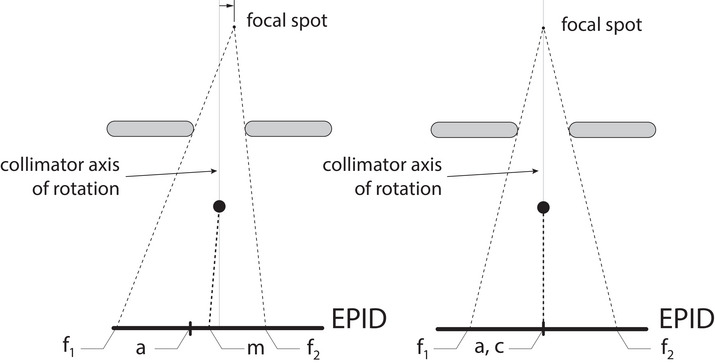
Schematic of how the EPID‐derived beam axis and radiopauqe marker position can be used to reduce the focal spot offset creating a properly aligned beam. With a radiation‐opaque marker accurately placed on the collimator's axis of rotation, a non‐negligible offset as shown in (a) will cause the marker location (m) and the beam axis (a) to diverge. When the offset is eliminated as shown in (b), the marker location and beam axis will be coincident within the image. The beam axis (a) is determined from the center of the field edges (*f*_1_ and *f*_2_) for a well‐calibrated field, or from the average field center from a pair of images acquired at a 180° collimator offset with otherwise identical setup. EPID, electronic portal imaging detector.

With the radiopaque marker accurately located on the collimator's rotation axis, radiation transmission images of the marker within a field can be used to eliminate any focal spot offset and align the beam. The process, shown schematically in Figure 4, involves adjusting the LINAC's bending magnets so that the marker location, derived from the image, coincides with the beam axis, also derived from the image. Note that the beam axis location in the electronic portal imaging detector (EPID) image can be determined either from the center of a well‐calibrated field, or, if the field is not well calibrated, from the average field center derived from a pair of images acquired at a 180° collimator offset with otherwise identical setup.

To optimize the bending magnet current, multiple images are acquired with varying bending magnet currents, and marker‐to‐beam‐axis errors were computed for each image. A linear least‐squares fit can be used to compute the optimal current that aligns the marker position in the image with the beam axis.

**FIGURE 5:**
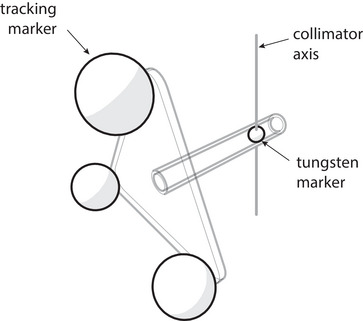
The target tracking tool contains three active tracking markers precisely positioned relative to a tungsten marker.

The marker is positioned on the found collimator axis with a special optical tracking tool shown schematically in Figure 5. This tracking tool contains a precise assembly of three optical tracking spheres relative to a tungsten radiopaque marker. This tool, referred to as a *target tracking tool*, can be attached to a XYZ linear adjustment stage so that the position of the tungsten marker can be adjusted in real‐time under optical guidance.

### 2.6 Couch axis alignment

The stability of the physical isocenter makes it a natural landmark for couch alignment, which we performed also using the isoPoint optical tracking system. To measure the couch axis of rotation, the gantry is maintained stationary and the couch is rotated while the camera pod tracks the pointer tracking module. This produces apparent movement of the pointer relative to the camera.

The optical tracking data of the pointer's apparent movement are used to compute the couch axis of rotation, using the same technique described above for the collimator axis determination. To ensure that both the couch axis and the physical isocenter are in the same coordinate system (if they are to be compared), the couch axis is measured first and then the iMarc dataset is collected while the couch and, crucially, the camera pod positioned on it remain fixed.

A couch position error vector can be computed with Equation (2), where in this case, *p* is the physical isocenter location and A is the couch's axis of rotation. Couch adjustments can then be performed to minimize the error vector, converging the couch axis toward physical isocenter.

### 2.7 Marker placement

Once the couch alignment and radiation beam alignment are complete, the target tracking tool is optically guided to accurately position the tungsten marker at physical isocenter. With the marker at the physical isocenter, the results of any WL type test will be within a stable and reproducible reference frame.

### 2.8 Demonstration of technique

A simple demonstration is presented here to provide a working example of the methods described above. The goal of the demonstration is to show that with a simple optical tracking system, the LINAC subsystems that contribute to geometric accuracy can be individually optimized without the use of the WL technique. The technique is demonstrated in two parts:

- Marker shifts due to couch rotations before and after couch axis alignment to physical isocenter.
- Congruence of beam axes to collimator axes of rotation before and after beam alignment.

**FIGURE 6:**
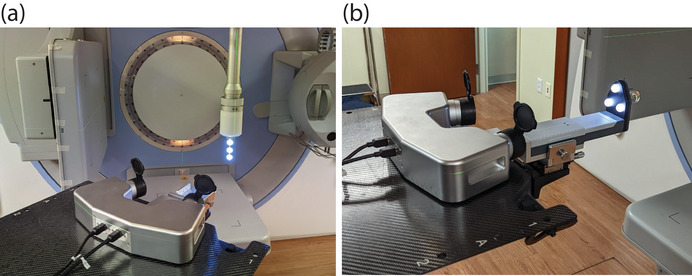
The Aktina Medical isoPoint system showing (a) the pointer tracking tool connected to the LINAC collimator and (b) the target tracking tool connected to the end of the couch top via a precision XYZ positioning stage. Also shown in both views is the stereo‐vision camera pod.

A prototype version of the Aktina isoPoint system running software version 2023.0 (Aktina Medical Corp., Congers, NY, USA) implements the techniques described in this paper. The main components of the isoPoint system are a stereo‐vision camera pod, a pointer tracking tool, and a target tracking tool shown in Figure 6. This system is capable of measuring tracking tool displacements with 0.1% accuracy and 0.01‐mm precision. The system acquired tracking data at approximately 100 data points per full collimator rotation. All data were acquired on an Elekta Infinity LINAC (Elekta AB, Stockholm, Sweden).

To show the reproducibility of the collimator axes for computing physical isocenter, five iMarc datasets were acquired and the standard deviation of the location and radius were computed.

For couch alignment, optical data for both clockwise and counter‐clockwise couch rotations were acquired and the average of the two axes was aligned to the physical isocenter. This work was performed on a LINAC during installation before the couch was initially aligned by the installer. To demonstrate the effectiveness of the alignment, marker shifts due to couch rotation were computed. With the couch at 0°, the marker was first positioned to physical isocenter accurately using optical guidance. EPID images were then acquired at the couch angles of $-90^{\circ }$, $-45^{\circ }$, 0°, 45°, and 90°. The marker shifts were computed as the difference between the marker center for nonzero couch angles relative to the couch $-0^{\circ }$ marker center. Marker centers were determined from each EPID image as the centroid of the marker based on the markers 50% contour, projected from the EPID to isocenter.

Beam alignment was demonstrated at gantry angle 0° along the in‐plane direction using the bending‐fine current control, which the Elekta control system applies globally across all gantry angles. Once the marker was positioned on the gantry 0° collimator axis (measured with optical tracking), the bending‐fine current was adjusted from 1.7 A through 2.1 A in 100‐mA steps. For each bending‐fine value, pairs of images with 180° collimator separation were acquired with 50MU. The field centers were determined from each EPID image as the centroid of the field 50% contour.

A linear fit of the in‐plane beam‐axis‐to‐marker distance versus bending‐fine value was used to solve for the bending‐fine value that produced in‐plane coincidence between the marker and the beam axis.

After the optimal bending‐fine value was computed and entered into the LINAC control system, the marker was optically guided to the physical isocenter and eight EPID images were acquired at the four cardinal gantry angles (two 180° collimator opposed images each gantry angle).

The beam axes were determined from the average of the field centers relative to the marker from each image pair. A beams‐eye‐view composite plot relative to physical isocenter of all collimator axes of rotation and radiation beam axes were created to visualize the results of the in‐plane beam alignment that was performed. This type of axis‐by‐axis comparison plot was chosen over a typical radiation isocenter analysis since radiation isocenter can obscure the behavior of the underlying beam axis. By comparing the radiation beam axes to their corresponding collimator axes, we are able to obtain a fine‐grained understanding of how the LINAC is performing across different gantry angles.

## 3 RESULTS

### 3.1 Physical isocenter

**FIGURE 7:**
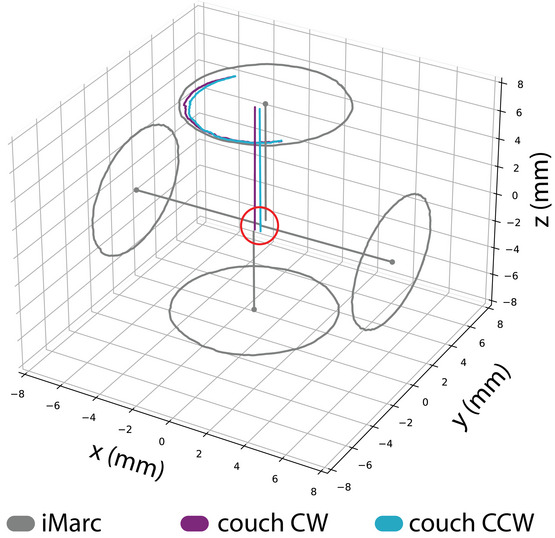
Optical tracking data and computed collimator axes of rotation used to determine physical isocenter (shown in gray). The red sphere designates the location and size of physical isocenter, which was found to have a radius of 0.60 mm. Couch tracking data and computed couch axes of rotation (for both CW and CCW couch rotations) prior to couch alignment are also shown.

**FIGURE 8:**
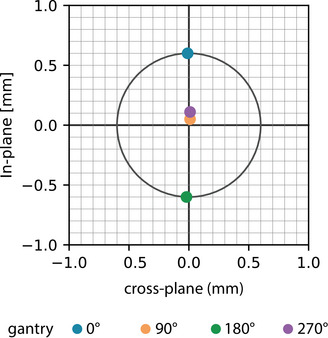
Superimposed beams‐eye‐views of the collimator axes of rotation relative to the found physical isocenter location (origin). The outer circle designates the size of the physical isocenter.

The acquired iMarc tracking data with computed collimator axes and the resulting physical isocenter are shown in Figure 7. The collimator axes for this dataset are also shown in a beams‐eye‐view coordinate system in Figure 8. As expected, the in‐plane shift of the gantry between angles 0° and 180° dominate the physical isocenter radius determination.

The five iMarc measurements resulted in a physical isocenter position standard deviation of 2.7 × 10^−3^ mm and a radius average of 0.60 mm with a standard deviation of 3.0 × 10^−3^ mm.

### 3.2 Couch alignment

**FIGURE 9:**
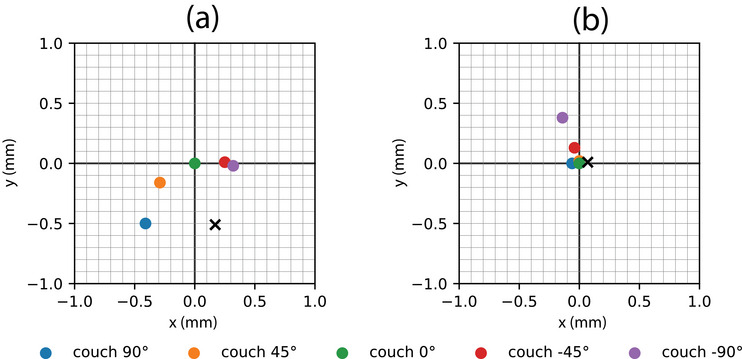
Marker position versus couch angle relative to physical isocenter for before couch alignment (a) and after couch alignment (b). The couch axis (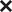) to physical isocenter coincidence error, as measured with optical tracking, before and after alignment were 0.54 mm (*x* = 0.17, *y* = −0.51) and 0.07 mm (*x* = 0.07, *y* = 0.01), respectively. The mechanical characteristics of the couch produces an outlier at $-90^{\circ }$.

Tracking data used to compute the couch axis prior to couch alignment are shown in Figure 7 for both clockwise and counter‐clockwise couch rotations. The average couch rotation was computed and determined to have a coincidence error with physical isocenter prior to couch adjustment of 0.54 mm (*x* = 0.17, *y* = −0.51). The couch‐induced marker shifts for the initial setup, determined from the WL images, are shown in Figure 9a.

The coincidence error vector between the couch axis and physical isocenter was used to compute height adjustments to the couch's front‐side tilt anchors, and a machinists dial gauge was used to accurately realize those adjustments. For each adjustment, a new iMarc dataset and a new couch rotation dataset were acquired. Two iterations were performed in approximately 20 min to achieve a couch axis to physical isocenter coincidence error of 0.07 mm (*x* = 0.07, *y* = 0.01). The couch‐induced marker shifts for the final aligned setup, determined from the WL images, are shown in Figure 9b.

We can see from Figure 9 that, aside from the outlier at couch $-90^{\circ }$, using optical tracking to position both the couch axis and the marker at physical isocenter results in marker shift magnitudes due to couch rotation less than 0.2 mm. The marker position at couch $-90^{\circ }$ in both subplots of Figure 9 indicates that a larger systematic couch rotation walkout error is occurring at this couch position.

### 3.3 Beam alignment

**FIGURE 10:**
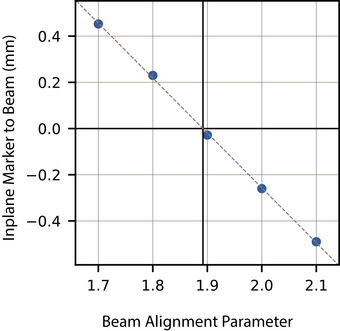
In‐plane marker error versus bending‐fine value used to computed the optimal bending‐fine value. The linear least‐square fit is shown (*m* = −2.375, *b* = 4.494), the optimal value was determined to be 1.89.

**FIGURE 11:**
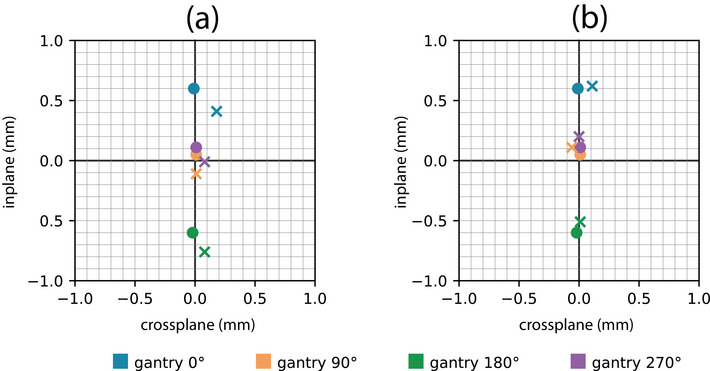
Superimposed beams‐eye‐views of the collimator axes of rotation (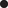) and the radiation beam axes (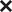) before beam alignment (a) and after beam alignment (b). All positions are shown relative to the physical isocenter. The initial bending‐fine beam current value was 1.95 and the optimized value was 1.89.

The measured in‐plane marker‐to‐beam distances versus bending‐fine values are shown in Figure 10. The optimal bending‐fine value was determined to be 1.89. Radiation beam axes superimposed over the collimator axis are shown in Figure 11, for both the initial bending‐fine value of 1.95 (subplot A) and optimal value of 1.89 (subplot B). The average collimator to beam axis distance before and after beam alignment was 0.19 and 0.10 mm, respectively.

## 4 DISCUSSION

We have presented a framework for measuring, tracking, and tuning isocenter. The foundation of this framework is physical isocenter, based on the mechanical collimator axis of rotation, which establishes a reference frame in which other LINAC properties such as couch position and beam alignment can be tuned.

The stability of a reference frame based on physical isocenter depends on the reproducibility of the collimator axis of rotation at each gantry angle. Even though the collimator axes have been reported to be reproducible,^11^ there is little published data on long‐term collimator stability. The day‐to‐day and long‐term stability of a reference frame based on physical isocenter is a natural next step for this work. Such studies would require an additional independent stable reference to assess physical isocenter against. Fortunately, the techniques described here could be used to detect changes in physical isocenter over time. For example, the relative position of the collimator axes shown in Figure 8 could establish a baseline for routine physical isocenter consistency checks. Furthermore, the gantry‐dependent collimator rotation tracking data (shown in Figure 7) can be used for collimator rotation walkout QA.

We used an optical tracking system to determine the mechanical collimator axes from which physical isocenter is determined. We used the same optical tracking system for couch adjustment and beam alignment, converging the couch axis and radiation isocenter to the physical isocenter. The optical tracking system was also used to accurately position a radiopaque marker to physical isocenter in order to compare radiation beam axes to collimator axes across gantry angles.

For simplicity, we demonstrated beam alignment using the Elekta bending‐fine control to reduce beam‐to‐collimator axis errors. This was used as first‐order correction across all gantry angles. More complex implementations are also possible within this framework.

For example, gantry angle‐specific cross‐plane beam alignment could also be implemented on an Elekta LINAC for flattening‐filter‐free (FFF) beams. The 2T lookup table values across gantry angles can be determined with the techniques described here. This would be effective at controlling the cross‐plane beam alignment as the gantry rotates since the 2T cross‐plane symmetry servo is off for FFF beams. Without the servo on, the lookup table entries become the final set point values (instead of only the initial values before the servo adjusts the magnet current to maintain symmetry). The Elekta LINAC allows for additional control since it permits unique 2T lookup tables for each FFF energy.

A marker at physical isocenter also allows for on‐board imaging to be aligned to physical isocenter as well, completing the realization of physical isocenter as the landmark for the couch, radiation, and imaging LINAC subsystems.

By encapsulating the mechanical collimator axes in the physical isocenter, and excluding the couch and other system elements, a natural modular structure emerges, in which the physical isocenter forms a stable target for aligning the couch, as well as optimizing the radiation isocenter.

We advocate the term *physical isocenter* to describe an isocenter derived from the collimator axes, rather than the term *mechanical isocenter*, which has been associated with a variety of definitions in the past, and is traditionally incorporates the gantry axis as well as collimator axes.

We believe that some of the techniques presented here may benefit from further development and refinement in future work. In the present work, we focused primarily on providing a working example of the methodology using a prototype optical tracking system. We plan to study the accuracy and repeatability of this technique using the final commercially available system. Future works will develop practical workflows for using these methods to fully optimize LINACs across all vendors. Additional research could explore alternate cost functions and optimization techniques beyond the expedient and simple cost function used here. We also expect that investigations into improved couch models across different LINACs will lead to increased treatment accuracy.

## 5 CONCLUSION

We have defined *physical isocenter* based purely on the collimator axes of rotation, excluding sources of drift such as couch, beam alignment, and field shaping. The physical isocenter replaces the traditional mechanical isocenter and serves as a stable target for the radiation isocenter and for couch axis alignment. We have presented methods for beam alignment and couch axis alignment that do not rely on the WL technique, allowing for modular tuning of the LINAC subsystems that contribute to geometric beam‐to‐tumor treatment accuracy.

We demonstrated the effectiveness of this method by presenting a typical isocenter optimization case, which was performed quickly and accurately with available technology.

## AUTHOR CONTRIBUTIONS

Nicholas G. Zacharopoulos conceptualized the framework and implemented the technology required for the manuscript. David A. Fenyes provided technical guidance. Nicholas G. Zacharopoulos was primary in composition of the manuscript. Both authors substantially contributed to the editing and interpretation of the content.

## CONFLICT OF INTEREST STATEMENT

The authors are employed by Aktina Medical Corp.

## Data Availability

The data that support the findings of this study are available from the corresponding author upon reasonable request.

## References

[1] Smith K , Balter P , Duhon J , et al. AAPM Medical Physics Practice Guideline 8.a.: linear accelerator performance tests. J Appl Clin Med Phys. 2017;18:23‐39. 28548315 10.1002/acm2.12080 PMC5874895

[2] Skworcow P , Mills J , Haas O , Burnham K. A new approach to quantify the mechanical and radiation isocentres of radiotherapy treatment machine gantries. Phys Med Biol. 2007;52:7109‐7124. 18029996 10.1088/0031-9155/52/23/022

[3] Chojnowski JM , Sykes JR , Thwaites DI. A novel method to determine linac mechanical isocenter position and size and examples of specific QA applications. J App Clin Med Phys. 2021;22:44‐55. 10.1002/acm2.13257 PMC8292690 34056850

[4] Nelder J. A simplex method for function minimization. Comput J. 1965;7:308‐313.

[5] Kanatani K , Rangarajan P. Hyper least squares fitting of circles and ellipses. Comput Stat Data Anal. 2011;55:2197‐2208.

[6] Nyiri BJ , Smale JR , Gerig LH. Two self‐referencing methods for the measurement of beam spot position. Med Phys. 2012;39:7635‐7643. 23231311 10.1118/1.4766270

[7] Slama LA , Riis HL , Sabet M , et al. Beam focal spot intrafraction motion and gantry angle dependence: a study of Varian linac focal spot alignment. Physica Medica: PM. 2019;63:41‐47. 31221407 10.1016/j.ejmp.2019.05.021

[8] D,. Létourneau, Keller H, Becker N, Amin MN, Norrlinger B, Jaffray DA. Quality control methods for linear accelerator radiation and mechanical axes alignment. Med Phys. 2018;45:2388‐2398. 29645282 10.1002/mp.12910

[9] Barnes MP , Menk FW , Lamichhane BP , Greer PB. A proposed method for linear accelerator photon beam steering using EPID. J Appl Clin Med Phys. 2018;19:591‐597. 30047209 10.1002/acm2.12419 PMC6123104

[10] Chojnowski JM , Barnes MP , Sykes JR , Thwaites DI. Beam focal spot position: the forgotten linac QA parameter. An EPID‐based phantomless method for routine Stereotactic linac QA. J App Clin Med Phys. 2017;18:178‐183. 10.1002/acm2.12147 PMC5875833 28786168

[11] Gibbs FA , Buechler D , Leavittph. d DD , Moeller JH. Measurement of mechanical accuracy of isocenter in conventional linear‐accelerator‐based radiosurgery. Int J Radiat Oncol Biol Phys. 1993;25:117‐122. 8416867 10.1016/0360-3016(93)90153-m

